# Range Expansion Drives Dispersal Evolution In An Equatorial Three-Species Symbiosis

**DOI:** 10.1371/journal.pone.0005377

**Published:** 2009-04-29

**Authors:** Guillaume Léotard, Gabriel Debout, Ambroise Dalecky, Sylvain Guillot, Laurence Gaume, Doyle McKey, Finn Kjellberg

**Affiliations:** 1 Centre d'Ecologie Fonctionnelle et Evolutive (UMR 5175), Montpellier, France; 2 Behavioural and Evolutionary Ecology, Université Libre de Bruxelles, Brussels, Belgium; 3 Institut de Recherche pour le Développement (IRD), UMR Centre de Biologie et de Gestion des Populations, Campus International de Baillarguet, Montferrier-sur-Lez, France; 4 Botanique et bioinformatique de l'architecture des plantes (UMR 5120), Montpellier, France; University of Arizona, United States of America

## Abstract

**Background:**

Recurrent climatic oscillations have produced dramatic changes in species distributions. This process has been proposed to be a major evolutionary force, shaping many life history traits of species, and to govern global patterns of biodiversity at different scales. During range expansions selection may favor the evolution of higher dispersal, and symbiotic interactions may be affected. It has been argued that a weakness of climate fluctuation-driven range dynamics at equatorial latitudes has facilitated the persistence there of more specialized species and interactions. However, how much the biology and ecology of species is changed by range dynamics has seldom been investigated, particularly in equatorial regions.

**Methodology/Principal Findings:**

We studied a three-species symbiosis endemic to coastal equatorial rainforests in Cameroon, where the impact of range dynamics is supposed to be limited, comprised of two species-specific obligate mutualists –an ant-plant and its protective ant– and a species-specific ant parasite of this mutualism. We combined analyses of within-species genetic diversity and of phenotypic variation in a transect at the southern range limit of this ant-plant system. All three species present congruent genetic signatures of recent gradual southward expansion, a result compatible with available regional paleoclimatic data. As predicted, this expansion has been accompanied by the evolution of more dispersive traits in the two ant species. In contrast, we detected no evidence of change in lifetime reproductive strategy in the tree, nor in its investment in food resources provided to its symbiotic ants.

**Conclusions/Significance:**

Despite the decreasing investment in protective workers and the increasing investment in dispersing females by both the mutualistic and the parasitic ant species, there was no evidence of destabilization of the symbiosis at the colonization front. To our knowledge, we provide here the first evidence at equatorial latitudes that biological traits associated with dispersal are affected by the range expansion dynamics of a set of interacting species.

## Introduction

Variation in parameters of the Earth's orbit around the sun has led to recurrent dramatic climatic shifts throughout its history [1], [2], [3]. Climatic shifts have in turn resulted in tremendous changes in the geographical distribution of species [4], [5], [6], [7], a phenomenon named Orbitally Forced species Range Dynamics (ORD) [8], [9]. ORD is a major evolutionary force, shaping many life history traits of species, and could govern global patterns of diversity through spatial variation in rates of extinction and speciation. Repeated and strong climatic shifts are predicted to favor more dispersive, generalistic and widespread species [8], [9]. Specialized narrow-endemic species are postulated to have persisted in equatorial regions because of weaker ORD. However, data on ORD in such regions are largely wanting, especially in the Paleotropics [10], [11].

Within species, vagility (i.e., ability and propensity to disperse) is predicted to be favored during range expansion episodes [12], [13]. Phenotypes presenting high vagility are more likely to found new populations and their offspring to preempt suitable habitats [14]. Indeed, within species, traits associated with vagility are often more developed close to colonization fronts [e.g. 15], [16], [17], [18]. A wide set of traits may be affected. For instance, in the shrub *Frangula alnus* young populations of central Europe differed from old populations in Spain in lifetime reproductive strategy, fruit morphology, fruit ripening phenology and, as a consequence, in their associated guild of seed dispersers [19]. These phenotypic changes may be transient, as documented e.g. in plants [20], insects [21] and amphibians [18]. Speed of reversal depends on the tradeoff between life history traits [17]. Hence, phenotypic modifications selected during colonization may in some species be preserved for many generations, bearing witness to historical or even ancient processes.

Another predicted consequence of ORD is reduced quantitative importance of co-evolutionary processes. Indeed, different species may expand at different rates, limiting opportunities for continued interactions. Hence strong ORD limits opportunities for long term co-evolution [8]. When associations are maintained, the co-evolutionary process should be affected by selective pressures specifically linked to ORD. Indeed, in associations involving horizontal transmission and formed anew each generation (e.g. lichens, mycorrhizae, most ant-plant protection mutualisms) sexual reproduction of any of the partners, with dispersal of diaspores, is at the expense of the other partner [for examples], [see 22], [23]. Therefore ORD, selecting for a higher investment in reproduction, should also select for less lavish mutualists and more virulent parasites.

Studies of ORD and its biological consequences within species have focused on temperate-zone species [5], [24], [25]. However, extreme populations of these species are generally located at their ecological limits, in zones of poor habitat quality, and therefore provide information on adaptation that mixes response to ORD and response to local habitat conditions. Further, because populations of temperate-zone species often cover huge surfaces, they are exposed to heterogeneous ecological conditions. It is thus difficult to separate clinal traces of historical responses to past ORD from phenotypic responses to spatial variation in current ecological conditions. On the other hand, under rather homogeneous abiotic conditions of equatorial regions, ranges of species could be limited by dispersal rather than by availability of favorable habitats. This pattern should be particularly pronounced in low-dispersal species, for which strong genetic differentiation is expected over short distances. Such species would provide ideal biological models to monitor the biological signatures of an ORD-driven progression over short distances. When compared with results from temperate regions, studies of equatorial and low-dispersal species may thus give original, independent and complementary information on consequences of ORD.

We investigate here ORD and its influence on traits associated with dispersal in a narrowly endemic three-species symbiosis of African equatorial forests. The tree *Leonardoxa africana* subsp. *africana* is an ant-plant, which provides its mutualistic ant *Petalomyrmex phylax* with nest sites in its hollow internodes and feeds it nectar. A numerous worker force continuously patrols and efficiently protects young leaves against insect herbivores [26]. Each tree is occupied by a single ant colony. *Cataulacus mckeyi* is an ant that may replace *P. phylax* and that is parasitic on this mutualism. It feeds on the nectar, uses the hollow internodes but has much smaller numbers of patrolling workers, which confer little or no protection against herbivores [27]. We show that the three species present congruent genetic signatures of recent gradual southward expansion of the symbiosis. This expansion is compatible with available regional paleoclimatic data. Further, in agreement with theoretical predictions, we show that the two ant species present, for a whole suite of traits, more dispersive strategies close to the colonization front. Hence we demonstrate that ORD is relevant to understanding the distribution and biology of species even in one of the regions where it is least expected and where it is most debated. At the verge of a dramatic climate shift episode triggered by human-induced global change, exploring how species responded to past ORD in the places where they are predicted to be most sensitive may help us anticipate consequences of future climate change.

## Results

### Genetic signature of a north-to-south postglacial expansion

**Figure 1:**
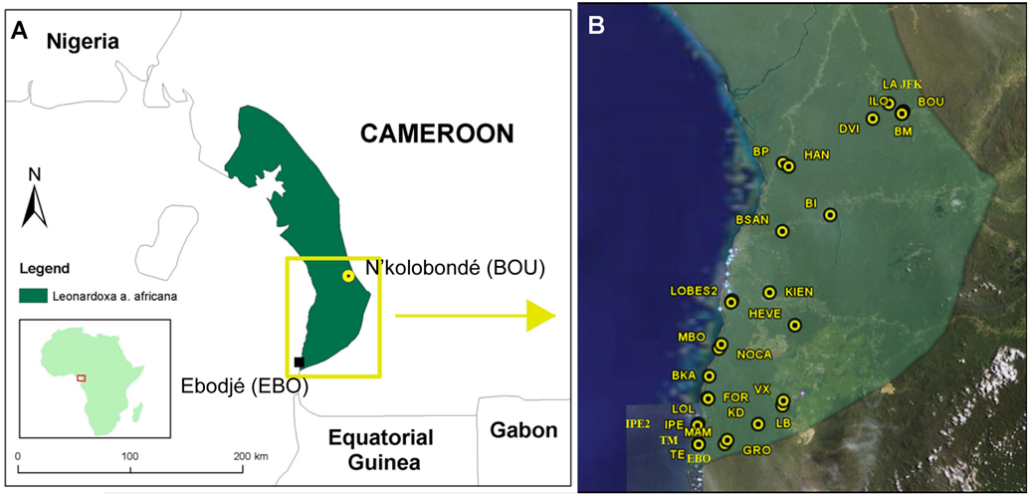
The study area. A. Distribution of the host-plant *Leonardoxa a. africana*. B. Location of the 29 populations of trees and associated ants that were sampled.

**Figure 2:**
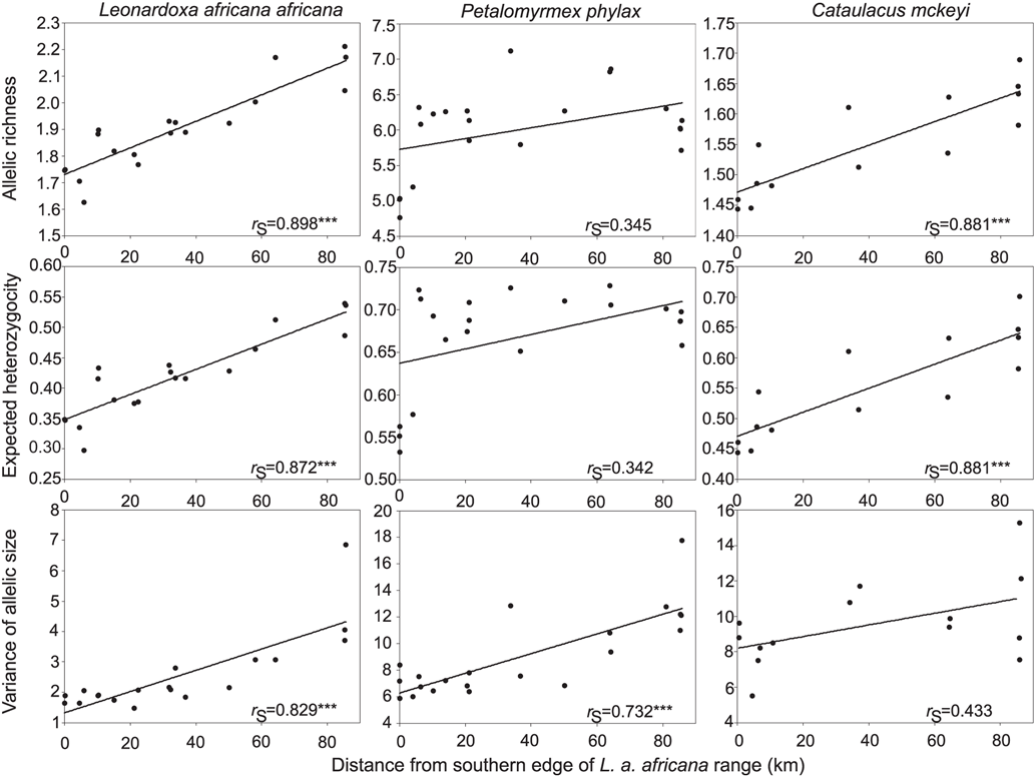
Genetic signatures of southward range expansion in the three species association. Geographic clines for three measures of within-population genetic variation at microsatellite loci of the host-plant *Leonardoxa a. africana*, the mutualistic ant *Petalomyrmex phylax* and the parasitic ant *Cataulacus mckeyi*.

Within the southern third of the historic range of the system (Fig. 1) we investigated genetic diversity within and among populations for each of the three species, in order to test for recent southward range expansion of the symbiosis. The three taxa presented a consistent pattern of reduced within-population genetic diversity from north to south. This pattern was marked for gene diversity, *H_E_*, variance of allele size, *V*, or both (Fig. 2). For the host plant, *L a. africana*, both measures of genetic diversity decreased strongly and significantly towards the postulated colonization front. For the mutualist ant *P. phylax*, clinal loss of diversity was significant for *V* only. However, the four very southernmost populations showed a strong decrease of *H_E_*, suggesting founder effects. Finally, the ant parasitic on the mutualism, *C. mckeyi*, exhibited a strong and significant clinal decrease of *H_E_*, while *V* values only presented a trend towards reduction southward. The global pattern of a north-to-south reduction in genetic diversity, congruent in all three species, is suggestive of a north-to-south colonization route.

Genetic differentiation among populations was marked for each of the three taxa. The global multilocus *F*_ST_ values were 0.109 for *L a. africana*, 0.126 for *P. phylax* and 0.170 for *C. mckeyi*. Differentiation between pairs of populations was significant for most comparisons for each of the three taxa (after correction for multiple tests, 161 out of 171 pairwise *F*_ST_ values were significant for the host plant *L. a. africana*; 203 out of 210 for the mutualist ant *P. phylax*; and 73 out of 91 for the parasitic ant *C. mckeyi*). Indeed, *F*_ST_ values between populations located less than two kilometers apart often revealed significant genetic differentiation, and this for the three species. Finally, a significant genetic pattern of Isolation By Distance (IBD) was detected for each of the three taxa (all *P*<10^−4^; linear regression with respectively intercept/slope/r^2^: −0.16/0.025/0.264 for *L a. africana*, −0.20/0.033/0.31 for *P. phylax* and −0.32/0.052/0.469 for *C. mckeyi*). Thus, overall differentiation among populations was strong and congruent in all three species, even at short spatial distances, suggesting spatially limited dispersal.

As we had three repeats of the same history, we inferred a rough estimate of the age of the cline using the difference in behavior among microsatellite markers. The effect of stepwise mutations on population divergence at a locus is predicted to be negligible for a divergence time *T*≪1/*μ* and to become apparent for *T*≥1/*μ* [28]. Three loci for *P. phylax* (out of 12), and only one for *L. a. africana* (out of nine) and for *C. mckeyi* (out of 10) presented statistics of population differentiation based on allele size, *R*_ST_, significantly higher from those based on allele identity, *F*_ST_. Divergence among populations is thus probably recent, as stepwise mutation at microsatellite loci contributed to differentiation between populations for only five out of 31 loci. These loci were used to estimate upper bounds of the confidence intervals for dating colonization of the transect, and the other loci were used to estimate the lower bounds. Estimates of the time of divergence in numbers of generations, *T*, were of the same order of magnitude for the three taxa: 968≤*T*≪3,104 for *P. phylax*, 712≤*T*≪2,503 for *C. mckeyi*, and 619≤*T*≪2,774 for their host-plant. Age estimates of the cline, expressed in numbers of generations, are rather similar, although the plant presents much longer generation time than the ants. We suggest that because more polymorphic loci, presenting higher mutation rates than average, were selected during marker development, the calculated upper bounds of the ranges may be overestimates. Whether the homogeneity in the estimates results from a non-random choice of highly polymorphic microsatellites during marker development requires further studies.

Overall, the combination of clinal loss of genetic diversity coupled with a genetic pattern of IBD and with recent divergence times between populations for the plant as well as its associated ants, strongly support the hypothesis that the whole system experienced a progressive and relatively recent range expansion towards the south.

### Multiple evidence for clinal variation in dispersal ability and the mutualism/parasitism continuum in the ants

We investigated variation of phenotypic traits implicated in species interactions that are likely to be selected during range expansion. For the plant we analyzed size at first reproduction and number of foliar nectaries. For the ants we analyzed investment in reproduction versus investment in colony survival, temporal dynamics of occupation of nest sites and variation in traits correlated with capacity to found new colonies.

#### (i) The ant-plant

The host plant *Leonardoxa a. africana* showed no evidence of a shortened juvenile period towards the south (Table 1). Indeed, the probability of having flowered at least once was correlated strongly with size of trees, but not with spatial distance from the southernmost occurrence of the species, nor with the interaction between tree size and spatial distance. Note that results were similar when the non-significant interaction term was removed from the models. The estimated size at which half the sampled trees had reached sexual maturity varied slightly among populations (from 17 to 26 mm for basal trunk diameter and from 1.7 to 2.5 m for tree height; Table S2) but did not show clinal variation along the transect (basal trunk diameter: *P* = 0.872, *r*_S_ = −0.086, *N* = 6 populations; tree height: *P* = 0.913, *r*_S_ = 0.058, *N* = 6 populations).

**Table 1:** No geographic cline in host-plant probability of having already flowered at a given size.

Effect	*b_yi_*±SE	*t*	*P*
Trunk diameter	0. 2658±0. 0355	7.486	<10^−4^
Spatial distance	0.0269±0.0143	1.880	0.060
Diameter * spatial distance	−0.0011±0.0007	−1.679	0.093
Intercept	−5.8556±0.8241	−7.105	<10^−4^
Regression: χ^2^_2_ = 293.84, *P*<10^−4^; McFadden's Rho^2^ = 0.527			

Effect	*b_yi_*±SE	*t*	*P*
Tree height	1.9021±0.2763	6.885	<10^−4^
Spatial distance	−0.0044±0.0114	−0.384	0.701
Height * spatial distance	0.0005±0.0066	0.082	0.935
Intercept	−3.2991±0.5157	−6.397	<10^−4^
Regression: χ^2^_2_ = 247.92, *P*<10^−4^; McFadden's Rho^2^ = 0.445			

Results of multiple logistic regressions explaining the probability of *L. a. africana* having reached sexual maturity as a function of the geographic distance to the southern edge of the range of the *Leonardoxa* system, controlling for the effect of tree size (measured either as diameter at the trunk base or as total height). χ^2^_2_: likelihood-ratio statistic of the model, *b_yi_*: partial regression coefficient (±Standard Error), *N* = 402 trees from six populations (see Table S2 for details on samples).

The mean number of observed nectaries per leaf varied little among populations (from 11 to 14) and did not show clinal variation along the transect (*P* = 0.948; *r*_S_ = 0.021, *N* = 12 populations). Hence there was no evidence for a clinal variation in food resources available to ants (see Table S3 for details). The same result was found when we considered the mean number of nectaries per basal leaflet (*P* = 0.101; *r*_S_ = −0.497, *N* = 12 populations). Thus, this component of investment of trees in production of food for the mutualists seemed to be independent of geographic location along the north/south transect. Hence, we detected no evidence of change in the host plant's phenotype in response to the colonization process.

#### (ii) The plant-ants

In both ant species, multiple regressions explaining the production of individuals of the different castes gave very similar results (Table 2). The main effect was that all parameters of colony productivity increased strongly with colony size. Further, we found for both ant species, *P. phylax* and *C. mckeyi*, significant and substantial partial correlations between distance to the southern edge of the distribution and production of both female sexuals and workers. Similar sized colonies produced more sexual females and fewer workers in the south than in the north. In contrast, male production remained constant. Hence our data showed an increasing numerical investment of colonies in female dispersal and a correlated decreasing investment in colony growth and maintenance towards the south for both ant species.

**Table 2:** A geographic cline in ant colony production parameters.

Worker production	*P. phylax* (F_2,65_ = 61.46***; adjusted R^2^ = 0.644)				*C. mckeyi* (F_2,58_ = 43.54***; adjusted R^2^ = 0.586)			
Effect	*b_yi_*±SE	*b_yi_′*	*T*	*P*	*b_yi_*±SE	*b_yi_′*	*T*	*P*
Size of colony	0.7674±0.0723	0.7755	10.608	<10^−3^	0.7215±0.0903	0.6694	7.990	<10^−3^
Distance	0.0013±0.0005	0.1836	2.511	**0.015**	0.0031±0.0008	0.3105	3.706	**<10^−3^**
Intercept	−0.1205±0.2527	–	−0.477	0.635	0.0444±0.2082	–	0.213	0.832

Female production	*P. phylax* (F_2,65_ = 12.85***; adjusted R^2^ = 0.261)				*C. mckeyi* (F_2,71_ = 14.16***; adjusted R^2^ = 0.265)			
Effect	*b_yi_*±SE	*b_yi_′*	*T*	*P*	*b_yi_*±SE	*b_yi_′*	*T*	*P*
Size of colony	0.8823±0.1856	0.5002	4.753	<10^−3^	0.7591±0.1593	0.4824	4.766	<10^−3^
Distance	−0.0028±0.0013	−0.2187	−2.078	**0.042**	−0.0044±0.0015	−0.3008	−2.972	**0.004**
Intercept	−2.1904±0.6484	–	−3.379	0.001	−0.9757±0.3643	–	−2.678	0.009

Male production	*P. phylax* (F_2,65_ = 14.06***; adjusted R^2^ = 0.281)				*C. mckeyi* (F_2,71_ = 9.01***; adjusted R^2^ = 0.180)			
Effect	*b_yi_*±SE	*b_yi_′*	*T*	*P*	*b_yi_*±SE	*b_yi_′*	*T*	*P*
Size of colony	1.4773±0.2787	0.5506	5.301	<10^−3^	0.8277±0.1954	0.4527	4.235	<10^−3^
Distance	−0.0004±0.0020	−0.0196	−0.189	**0.851**	−0.0005±0.0018	−0.0278	−0.260	**0.796**
Intercept	−4.3828±0.9733	–	−4.503	<10^−3^	−1.2452±0.4470	–	−2.786	0.007

Results of multiple regressions explaining the parameters of productivity of colonies of the mutualist ant *Petalomyrmex phylax* and the parasitic ant *Cataulacus mckeyi* for each caste (workers, winged females, males), as a function of the geographic distance (km) to the southern edge of the range of the *Leonardoxa* system when controlling for colony size (the number of adult workers). *b_yi_*: partial regression coefficient (±Standard Error), *b_yi_′*: standardized partial regression coefficient. *N* = 68 colonies for *P. phylax* and 61–74 for *C. mckeyi*.

Capture-recapture models applied to change in species occupancy on marked trees over three to seven years indicated a negative correlation between distance to the southern limit of the cline and turnover of inhabitants in *C. mckeyi*-inhabited trees (*P* = 0.019; *r*_S_ = 0.886, *N* = 6 populations), but failed to detect clinal variation of turnover of inhabitants in *P. phylax* inhabited trees (*P* = 0.623; *r*_S_ = 0.257, *N* = 6 populations, see Table S4). We suggest that, in *C. mckeyi*, the increased resource allocation to reproduction towards the south, and the associated reduction in worker production, translate directly into reduced colony survival.

**Figure 3:**
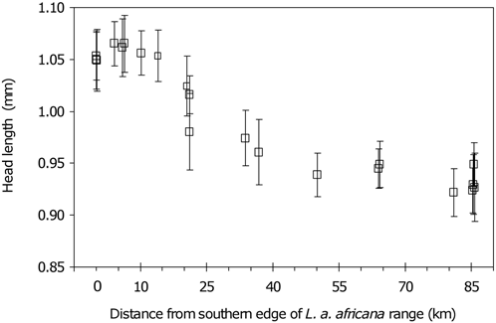
Body size of female sexuals of *P. phylax* increases southward. Geographic cline of within-population variation (mean±standard deviation) of head length, a surrogate of queen size, measured over 1,036 individuals from 256 colonies in 21 populations of the ant *P. phylax*.

In addition to variation at the colony level, *P. phylax* exhibited traits that varied clinally along the transect at the individual level. These were traits associated with body size of female sexuals. Head size, body weight, and wing size of female sexuals of *P. phylax* increased from north to south (for all tests *P*<0.001; −0.964≤*r*_S_≤−0.705; *N* = 21 populations; Fig. 3, see Table S5 for detailed data). Two geographically extreme populations of *P. phylax*, one at each end of the transect, EBO (the southernmost) and BOU (among the northernmost) were more thoroughly analyzed. Estimates of relative dry weight of adult females, but not that of adult males or workers, differed markedly between these two extreme populations (Table S6). The difference in female size was thus not a byproduct of a general north-to-south increase in ant size. Finally, the survival of *P. phylax* females after 60 days in conditions of claustral foundation (see Materials and methods) increased from north to south (*P* = 0.003; *r*_S_ = −0.827, *N* = 10 populations) (see Table S5), and was positively correlated with mean alate female dry weight per population (*P* = 0.021; *r*_S_ = 0.711, *N* = 10 populations). Thus, in *P. phylax*, size and weight of sexual females increased southward and translated into higher capacity to found new colonies.

## Discussion

Our genetic results provide strong evidence for recent southward range expansion of each of the three interacting species. Associated with this range expansion, changes in a set of traits involved in colonization dynamics were documented for both ant species and an investment tradeoff between sexual reproduction and worker force production was shown. As number of workers translates into plant protection potential and as each tree hosts a single ant colony, increased production of female sexuals when moving southwards may translate into reduced efficiency of the mutualism and higher virulence of the parasitism in terms of plant protection, increasing the conflict of interest between the symbiotic partners. However, the net outcome for the host plant's protection depends also on other traits beyond numerical investment in workers and queens, for instance worker activity rhythm, predatory behavior and recruitment dynamics [29]. Despite a potential for decreased mutualistic efficiency or increased virulence southward in the two ant species, available evidence from two geographically extreme populations along the transect suggests that ant behavioral traits may not vary enough among sites for this potential to be expressed [29]. The resulting effect of within-species variation in ant social structure on plant protection is an area for future research. Our preliminary data on herbivory levels of *P. phylax* and *C. mckeyi* inhabited trees over five plant patches sampled from one to three years (1999–2001, [30]) indicate strong spatio-temporal variations, over a few hundred meters and from year to year, which suggests that fine-scale variations in phytophagous insect pressure may be a strong confounding factor. As a result, variations in the net outcome of the symbiotic interactions from the plant's perspective may be difficult to quantify. Nevertheless, we did not find here any evidence that a main component of the flow of resources provided by the tree to its ants is reduced southward, showing no evidence that ORD had marked destabilizing effects on the mutualism within this particular system. We propose that the very long generation time of the host tree may have had a major stabilizing effect. These results suggest that symbiotic systems themselves may, in some circumstances, be resilient in the face of climatic change that affects their constituent partners.

### Recent range expansion

In all three species we observed genetic isolation by distance and decreasing genetic diversity towards the south. This provides strong data supporting recent southward expansion of the association. Indeed, the three species are very tightly associated and their population-genetic patterns tell the same story. We aimed at estimating a rough age of population divergence using microsatellite data. Despite large differences in generation time between tree and ants, we obtained similar rough estimates of elapsed numbers of generations, which suggests a bias. Nevertheless, as stated above the bias is likely directional, so that numbers of generations are likely to be overestimated. This strongly suggests a postglacial expansion, as upper bound estimates of divergence times are hardly compatible with the hypothesis of an older, Pleistocene, expansion. Understanding the individual origin, age and phylogeographic history of currently interacting species is central to understanding the forces that have shaped community structure and species evolution [31]. The idea of using parasites (and mutualists) to help analyze host history [32] is validated in this case study.

During the late Pleistocene and the Holocene, the rainforest of Atlantic equatorial Africa experienced recurrent cycles of contraction and expansion, driven by changes in climate [see e.g. 33], [34], [35], [36], [37]. Two episodes of more humid and warmer conditions may have driven the expansion of the *Leonardoxa* system. The first one corresponds to the end of the last glacial maximum (12,000 years BP), and the second one followed an arid episode centered 3,000–2,500 years BP. The data on *C. mckeyi* with an estimate of divergence time along the transect ≪2,500 generations, and a colony mortality of at least 25% per year (Table S4), suggest that the cline originated after the more recent arid climate episode.

Although data and models of past vegetation suggest recent events of contraction-expansion of lowland rain forest in central Africa, the consequences of these climatic episodes on current within-species genetic structure have hardly been explored. Most studies in tropical Africa connecting genetic structuring with ORD have investigated afromontane species [38], [39], [40]. The very few data available for species of lowland rainforests such as shrews [41], mandrill [42] and the tree *Aucoumea klaineana* [43] suggest that climate-driven range expansion may be common in Atlantic equatorial Africa –including Cameroon. The present study provides congruent data for three species involved in symbiotic interactions that have shared a common history, and thus adds strong support to the controversial issue of the impact of past climatic oscillations on the current structuring of biodiversity in African lowland equatorial rain forests, supposedly one of the most stable ecosystems on earth [8].

### Evolution of dispersal strategies during range expansion

A prediction about evolutionary consequences of colonization processes is that selection favors more dispersive strategies on the colonization front [12]. This evolutionary trend has been documented for a few species subjected to range shifts [e.g. 15], [16], [17], [18], but to our knowledge never at equatorial latitudes and never at such a small scale as in our three-species system. *Petalomyrmex phylax* exhibits a gradient of gyny (number of nestmate egg-laying queens per colony) along the transect, from populations exhibiting secondarily polygynous mature colonies in the northern part of the study area to populations in the south, only 86 km distant, presenting almost exclusively monogynous colonies [44]. Previous genetic analyses at the colony and population scales have shown that secondary polygyny in *P. phylax* mostly results from lack of queen dispersal associated with intra-nidal mating, whereas monogyny occurs through outbred queens dispersing and founding new colonies independently from the assistance of workers [44]. Dalecky et al. [44] have shown that the variation in social structure of *P. phylax* –along with its associated mating system– correlates with a historical process corresponding to a progressive colonization of coastal southern Cameroon. Gyny is one of several life history traits classically associated with dispersal in ants [e.g. 45]. In ants in general, a lower queen number is associated with larger queens as a result of their contrasted reproductive and dispersal strategies [e.g. 45]. We have documented here variation at the individual level in a series of other associated traits that are predicted to be selected for on a colonization front, including body size of *P. phylax* queens and a component of their capacity to found new colonies. It is noteworthy that clinal variation in body size was expressed in females but not in males and workers, which do not participate in foundation of new colonies. We note that the effect of wing load ( = body mass per unit wing area) variation on dispersal capability of *P. phylax* queens needs further study (see [45] p. 12). Furthermore, we observed very similar geographic clines for sexual investment at the colony level and an investment tradeoff between sexual reproduction and growth/maintenance in the two ant species, as well as a geographic cline in colony turnover in the parasitic ant *C. mckeyi*.

All these traits are predicted to be associated with selection for dispersal in ants, and we thus propose that we effectively observed a selected north-south gradient of vagility in both ant species. Such a gradient may result from range expansion, but it could alternatively result from variation in the ecological conditions encountered by the ants. Given that the habitat of these symbiotic ants is typically restricted to their host plant, we searched for variation in plant traits. However, the phenotypic homogeneity of *Leonardoxa* along the transect, in terms of life history strategy and number of extrafloral nectaries per leaf –a principal resource on which the ants feed– suggests that ant colonies benefit from equivalent resources all along the transect. Further, a careful search for indicators of variation in environmental characteristics that could be associated with disturbance and colony turnover, such as the amount of light reaching the forest floor, percentage of trees with trunks that had been broken by disturbances, or the availability of empty nest sites, did not identify any habitat characteristic that varied clinally from north to south [44]. Hence we propose that the best explanation available for the north-south variation in ant traits is selection for dispersive phenotypes on the colonization front. These dispersive traits seem to be lost very slowly. We therefore suggest that these traits may involve relatively limited selective costs. Furthermore, low gene flow among populations, as evidenced by the strong genetic isolation by distance, must have limited subsequent colonization by less dispersive genotypes.

The clines in adaptive traits we observe here concern species moving out from a postulated refugium zone in a biodiversity hotspot. Within hotspots, in areas of marked elevational relief, species or assemblages of species may move very slowly during climatic oscillations [46] so that selection for dispersal will be very limited. Our suggestion is that as soon as species move out of such buffered zones selection for dispersal during range expansion or range shift becomes an active force, a feature which can be expressed as within-species selection and as species sorting [9]. Indeed, a global analysis of Upper Guinean endemic forest species gives a pattern in which relatively widespread endemics are ruderal while species with very restricted ranges are linked to particularly humid places, i.e., probably refugia [47].

### General conclusion

The progression of these species has most likely been slow. The distance between the northernmost historically documented populations and today's southern limit hardly exceeds 200 km. This should be compared with some temperate-zone species which have expanded over thousands of kilometers after the last glacial maximum. Although the three studied species may have moved extremely slowly, we have shown here that ORD, as a historical process, is highly relevant to understanding contemporary local biology of the symbiosis. The role of past, contemporary and future climate change in shaping the biology of species in general and of symbiotic interactions in particular should not be neglected.

## Materials and Methods

### Distribution of study system and population sampling

The distribution of the plant is restricted to a band, 250 km long and 80 km wide, of coastal rain forests of Cameroon, from southeast of the Cameroonian volcanic arc to near the border with Equatorial Guinea [48]. It is an understory plant of mature rainforests, where it grows in patchily distributed, well-defined stands of up to some hundreds of individuals, near rivers and wet sites [48], [49]. Given this natural history and the low water-holding capacity of the sandy to clayey-sandy soils of coastal rain forests [35], the distribution of the plant is predicted to be highly sensitive to fluctuations in water availability, rainfall, and seasonality, and must have been strongly affected by climate changes. On the basis of biogeography and a cladistic analysis of morphological characters in the *L. africana* complex, McKey [48] proposed that *L. a. africana* ‘colonized coastal forests, spreading southward’.

The northern part of the current distribution of *L. a. africana* has been strongly affected by agriculture and other human activities and there are few places where the species still survives. In contrast, in the south of its range *L. a. africana* is locally abundant, representing a characteristic element of the caesalpinioid-rich flora of coastal rainforest. This is compatible with the hypothesis that *L. a. africana* is such a slow colonizer that it has yet to reach the southern limit of its potential range. This would also be compatible with the very slow growth of the host plant, for which qualitative observations from marked trees followed for several years (1989–1997) suggest that they will not flower before reaching tens of years in age.

For this study 29 populations, constituting a roughly north-south transect, were sampled for genetic studies, phenotypic studies or both (Fig. 1, see also Table S1 for detailed coordinates). This transect covers the southern half of the range of *L. a. africana*, and the population EBO (near the village Ebodjé) is the southernmost known population of *L. a. africana*. Despite the abundance of the plant and its ant occupants in the vicinity of Ebodjé, neither field nor herbarium research led to the discovery of more southerly populations of *L. a. africana*. EBO was thus considered to represent the probable front of colonization of the system, and in the following all statistical treatments involving geographic distance along the transect are based on geographic distance (in km) from the southernmost documented occurrence of the three species (population EBO). Unless stated otherwise, to assess the existence of clinal variation of each measure, we tested for a correlation between the spatial distance to EBO and the mean values per population using Spearman rank correlation coefficient (*r*_S_). Correlations were tested using the software Analyse-it version 2.12 (Analyse-it Software, Ltd. http://www.analyse-it.com/) or the VassarStats web site for statistical computation (http://faculty.vassar.edu/lowry/corr_rank.html).

### Testing for recent range expansion

Several genetic features are expected to be signs of recent range expansion. During the period of colonization, each successive foundation of a new population induces a bottleneck, thus reducing genetic diversity progressively from the place of origin of the expansion towards the front of colonization [5], [50], [51]. Furthermore, repeated foundation events along a colonization front result in adjacent populations being genetically more similar than distant populations, a pattern termed isolation by distance (IBD) [51], [52]. The patchy distribution of populations of this symbiotic system should not only facilitate bottleneck effects, but also the appearance of a genetic pattern of IBD. Hence, to test the hypothesis that the whole system has undergone a recent range expansion, we investigated for each species: (1) the spatial pattern of genetic diversity; (2) the pattern of genetic differentiation between pairs of populations (IBD); and (3) the time elapsed since divergence of populations.

We investigated genetic variation in host plant, parasitic ant and mutualistic ant using nuclear microsatellite markers: (i) Genetic variation in *L. a. africana* was analyzed for 594 diploid individuals from 19 populations (mean = 31 individuals per population, range = 13–80) sampled in 2000–2007, and individual genotypes were inferred using 9 markers amplified following Léotard et al. [53]. (ii) Genetic variation in *C. mckeyi* was analyzed for 206 diploid individuals from 14 populations (mean = 15 individuals per population, range = 5–62, each from a different colony) sampled in 2000–2002, and genotypic data from 10 markers were already available for this sample [54]. (iii) Genetic variation in *P. phylax* was analyzed for 475 diploid individuals from 21 populations (mean = 23 individuals per population, range = 13–66, each from a different colony) sampled in 2000–2002, and genotypic data from 12 markers were already available for this sample [55]. Within species, all genetic markers were shown to be statistically independent with the program GENEPOP 3.3 [56].

Using the software SPAGeDi 1.1 [57], we computed for each taxon and population two estimators of diversity across loci, one based on allele identity, the expected heterozygosity *H*_E_ [58], and the other based on allele size, the variance of allele size *V* [59]. If the dynamics of return to equilibrium are driven by local mutations involving a stepwise mutation model, then *V* returns to equilibrium much more slowly than *H*_E_. Thus *V* is expected to enable detection of older (in terms of numbers of generations) colonization processes than is *H*_E_ [59], [60]. Three other allele identity-based estimators of diversity (*n*_p_, the number of polymorphic loci; *n*_a_, the mean number of observed alleles; and *A*, the mean allelic richness using the rarefaction method to provide unbiased estimates corrected for unequal sample size [61]) were computed using the program FSTAT 2.9.3.2. [62] and their mean values across loci per population are presented as supporting information (Table S1).

For each species, genotypic population differentiation was analyzed for each pair of populations and over all populations by estimating *F*_ST_ values following Weir and Cockerham [63], and tested using exact tests with the program GENEPOP 3.3 [56]. Tests of IBD were performed over all populations by regressing multilocus pairwise estimates of *F*_ST_/(1−*F*_ST_) on pairwise ln-geographical distances [64], and tested with the Spearman rank correlation coefficient using Mantel-like tests based on 10,000 permutations of locations with SPAGeDi.

In an attempt to produce a rough estimate of the age of the cline, we tried to estimate the mutation rate for each locus and then used the hypothesis of Hardy et al. [28] that the effect of stepwise mutations on population divergence will be negligible (*R*_ST_ = *F*_ST_) for a divergence time *T*≪1/*μ* generations and will become apparent (*R*_ST_>*F*_ST_) for *T*≥1/*μ* generations. First, the mutation rate at locus *i* was estimated as 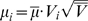 [65], where the average mutation rate of microsatellite loci, 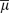, was set at 5×10^−4^ [e.g. 66], *V*_i_ was the observed average variance of allele size at locus *i* over all populations and 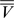 the average population mean (across loci) of the observed variance of allele size. Then to assess whether stepwise mutation at microsatellite loci contributed to differentiation between populations, statistics of population differentiation based on allele identity (*F*_ST_) and based on allele size (*R*_ST_) were compared following Hardy et al. [28], using SPAGeDi. Finally, for each species, we averaged the rate of mutation of the loci presenting a significant difference between *R*_ST_ and *F*_ST_ to compute a rough lower bound of the time elapsed since the divergence of populations, and we used the average rate of mutation of the other loci to compute a rough upper bound of this time.

### Testing for evolution of life history traits in the host-plant

Some north-temperate plants are known to reproduce earlier in life in sites in the northern part of their range, far from glacial refugia [19]. This is interpreted as the result of selection for dispersal on the colonization front. To test whether *Leonardoxa* reached sexual maturity at progressively smaller size when getting closer to the postulated colonization front we measured, for a total of 402 trees in six populations (41 to 126 trees per population), the diameter at the trunk base (in mm) and estimated the total height (in m). We then scored the stage of maturity (immature, i.e., that had never flowered yet, or mature, i.e., that had already flowered) of each individual. Indeed *Leonardoxa a. africana* is cauliflorous and the inflorescences leave detectable scars on the trunk, so that each tree could be categorized as immature versus mature. The relationship between the stage of maturity and geographical distance to the colonization front, controlling for the effect of tree size (measured either as diameter at the trunk base or as total height), was assessed using multiple logistic regression analysis with the program SYSTAT 9. Size of sampled trees and geographical distance were slightly correlated (all *P*<10^−3^; diameter: *r*_S_ = −0.346, height: *r*_S_ = −0.271, *N* = 402 trees), so we included an interaction term in the statistical model. For each population sample separately we then used multiple logistic regressions to estimate the size at which half the trees had reached sexual maturity and tested whether these estimates varied clinally.

To assess spatial variation in a main component of food resources provided by the plants to ant colonies, we counted the number of nectaries per leaf for 1,293 leaves (4–11 leaves per tree) from a total of 161 trees occupied by *P. phylax* in 12 populations (10–30 trees per population) in 2000–2002. *L. a. africana* possesses paripinnate leaves with four to eight leaflets. For this analysis missing leaflets were thus considered as bearing no nectaries. This analysis provides an estimate of this component of actual resources per leaf provided by the trees to the ants. In a separate analysis of the dataset, we quantified the mean number of nectaries produced per basal leaflet and we considered missing leaflets as missing data. This analysis provides an estimate of the potential, ontogenetic, investment of the plants in production of food per leaf for ant colonies. We only used the basal leaflets because the number of nectaries varies according to leaflet position, basal leaflets bear the greatest number of nectaries, and basal leaflets are less often missing than those in other positions.

### Testing for evolution of life history traits in the ants

#### (i) Colony productivity

It is predicted that, along a colonization front, organisms should invest more in reproduction and hence dispersal at the expense of survival. In both ant species we tested for variation among populations in the production by colonies of new workers, gynes and males. Eighty-six colonies from 13 populations for *P. phylax* and 75 colonies from 10 populations of *C. mckeyi* were entirely collected. In *P. phylax*, colonies become effectively sexually mature when reaching 1,000 workers [44]. Hence 68 colonies from 12 populations were retained for the analyses. Similarly for *C. mckeyi* the limit was set at 20 workers [54]. Hence 74 colonies representing 10 populations were retained. For each colony we counted the number of adults and the number of nymphs of each caste (worker, winged female, male). For each caste the productivity of the colony was computed as the number of young individuals in a cohort, and more precisely, the number of nymphs in *P. phylax* and the number of nymphs plus the number of young, not yet fully melanized imagos for *C. mckeyi*. Counting nymphs is intrinsically easier and in principle should yield a more precise estimate of investment, but numbers of nymphs were so low in *C. mckeyi* colonies that too much stochastic noise was added. Therefore the not yet fully melanized imagos (easily distinguishable in this heavily sclerotized species, in contrast to *P. phylax*) were very carefully counted, thus allowing the reduction of statistical noise in *C. mckeyi*.

The relationship between colony productivity and geographical distance to the colonization front, controlling for the effect of colony size (i.e., number of adult workers), was assessed using multiple regression analysis with the program SYSTAT 9 [67]. To ensure normal distributions of residuals, data were log transformed (Log_10_). The log-transformed variables were number of adult workers, number of young workers, (1+number of young males), (1+number of young females). To control for potential collinearity effects in multiple regressions, we verified that the explanatory variables –number of adult workers and geographic distance– were not correlated in this dataset (*P. phylax*: *P* = 0.404, *r*_S_ = 0.102, *N* = 68; *C. mckeyi*: *P* = 0.254, *r*_S_ = 0.134, *N* = 74). In multiple regressions, the partial regression coefficient (*b_yi_*) denotes the regression coefficient of each explanatory variable on the dependent variable, while removing the effect of all other explanatory variables as though they were kept constant. The standardized coefficient (*b_yi_′*) gives the rate of change in standard deviation units of the dependent variable per one standard deviation unit of explanatory variable, and allows direct comparison of the effects of the different variables independently of measurement units.

#### (ii) Temporal dynamics of tree occupation

The temporal dynamics of occupation of nest sites were investigated by monitoring a total of 1,151 permanently tagged trees in six populations (85 to 442 trees per population) over three to seven years. To describe population-level dynamics of the different occupancy states (unoccupied, occupied by *P. phylax*, by *C. mckeyi* or by both species), we used an information matrix derived from recapture histories of nesting sites and the multi-state option in the software MARK [68] that generates the maximum-likelihood estimates of annual survival and transition probabilities, Ψ. Each of the four occupancy states was assigned a different code, survival rate of nest sites (nest site = a tree) was fixed to 1 over the period considered, capture rate was constant and close to 1 (the value was determined through a first run of the program), the transition values Ψ were functions of the state but not of the time, and all Ψ that never happened within each site were fixed to 0.

#### (iii) Phenotypic traits of *P. phylax*: morphology and survival in conditions of claustral foundation of female sexuals

In *P. phylax*, foundation of new colonies, and hence colonization of new sites, depends solely on independent, claustral founding by winged females. In ant species that rely on independent foundation by a single winged female, colony founding ability is highly correlated with queen body size and weight [e.g. 45]. To investigate if the spatial gradient of gyny in *P. phylax* is linked to a gradient of dispersal ability, we assessed morphological variation by examining six interdependent traits that jointly estimate the relative size of adult queens: five morphometric estimates from two body parts (head and forewing) and the dry weight of entire individuals. For the five morphometric estimates, we collected data from a total of 1,036 adult female sexuals (10–376 per population) from a total of 256 colonies (2–53 per population) and 21 populations sampled in 1997–2003 and stored in 95% ethanol (for detailed numbers see Table S5). The head and forewing of each female, when available (head only for ‘dealate’ egg-laying queens) were mounted on double-sided adhesive tape to achieve a standard orientation and thus ensure precision of measurements. All measurements were made using an electronically assisted monocular lens (Nikon Measuroscope 10) under 30× magnification. Head length (HL) and head width (HW) refer to standardized measurements as in Hölldobler & Wilson [69, pp. 32–33]. Forewing measurements were as follows: total length (TWL), partial length (PWL) and total width (WW) (Fig. S1). In addition, dry weight was scored for 219 adult winged females (3–24 per population) from 176 colonies (1–20 per population) and 15 populations sampled in 2001–2002. Females were killed with ether vapor immediately when collected in the field, and then individually kept in boxes with desiccant (silica gel) to avoid dissolution of body constituents in ethanol. To achieve a standardized and thorough drying, individual females were dried for 24 h at 70–75°C and weighed. Preliminary analyses showed no significant effect of year of collection on the dry weight of 164 females collected in eight populations sampled in both consecutive years.

If female size primarily determines the success of foundation and dispersal, then we do not expect males and workers to follow the same pattern of spatial variation. To control the spatial variation of worker and male size we collected in March 1996 and stored in 95% ethanol a sample of 14–101 adult individuals per population from each caste (workers, alate females and males) from 10 colonies in population EBO (Ebodjé) and from 9 colonies in population BOU (N'kolobondé), two geographically extreme populations along the transect. These 270 individuals were removed from ethanol six months after collection, dried for 4 h at 40°C and weighed individually. Storage in ethanol dissolves some body constituents, such as lipids, so that these data are clearly underestimates but provide relative within-caste estimates of dry weight for these two populations. To test for differences in these relative estimates between these two populations, we performed nested analyses of variance for each caste separately with colony (within population) and population as factors explaining weight variation.

To further examine if female size is really determinant for successful foundation of a new colony, we conducted an experimental test of among-population variation of claustral founding capacity. In *P. phylax*, colony foundation is initiated by a winged female that disperses from her natal tree, searches for a vacant host-plant, digs a hole in a young twig, removes the pith from its inside, enters the domatium and blocks the entrance hole. The new queen thus confined within a domatium rears the first brood in isolation by claustral founding. To simulate claustral founding, a total of 146 winged females were collected inside colonies between March 30 and April 8, 2002 from ten populations (12–20 individuals from 7–10 colonies per population) (see Table S5). Single females were placed into 1.5 mL Eppendorf plastic tubes in which 25 holes (ca. 1 mm diameter) had been made in the bottom and wall of each tube to allow air circulation. Females were not fed as in natural conditions, and the bottom of each tube was filled with cotton and humidified as necessary to keep the cotton and the atmosphere moist. Tubes were randomly positioned in a dark ventilated chamber on a 13∶11 h cycle at 29∶26°C to mimic natural conditions. For each population, we computed the rate of survival of females after 60 days in conditions of claustral foundation, a typical duration for new worker development and emergence in ants [69], [70], [71], [72], [73], [74].

## Supporting Information

Table S1 The study populations: location and measures of genetic diversity. Statistics summarizing within-population genetic variation over 9 microsatellite loci for 19 populations of Leonardoxa a. africana, 12 loci for 21 populations of Petalomyrmex phylax, and 10 loci for 14 populations of Cataulacus mckeyi. Distance is the map distance (in km) from the southern edge of the range of the system. N is the number of diploid individuals genotyped per population; np, number of polymorphic loci; na, mean number of observed alleles; A, mean allelic richness; HE, mean expected heterozygosity; and V, mean variance of allele size. The two last rows indicate the means of values over all populations and the correlation between population means and the map distance from the southernmost limit of the range (Spearman rank correlation coefficient rS, asterisks indicate significant correlation [*: P<0.05, **: P<0.01, ***: P<0.001]). Data for P phylax are from Dalecky et al. [55].
(0.01 MB PDF)

Table S2 Size at which half the trees had reached sexual maturity. Estimated values [and 95% confidence interval] across trees per population and over all individuals of basal trunk diameter and tree height at which the probability of flowering is 0.50 based on logistic regressions. Populations are arranged in descending order according to their geographical distance from the southernmost known limit of the range of the system (these distances are given in Table S1). N is the total number of trees sampled, m is the number of sexually mature trees. The last row indicates the correlation between population estimates and the spatial distance from the southernmost limit of the range (Spearman rank correlation coefficient rS, all P not significant).
(0.00 MB PDF)

Table S3 Tree investment in the feeding of ants. Mean (±standard deviation) values across trees per population of number of (1) observed nectaries per leaf and of (2) produced nectaries per basal leaflet. Populations are arranged in descending order according to their geographical distance from the southernmost known limit of the range of the system (these distances are given in Table S1). N is the number of trees sampled. The last row indicates the correlation between population means and the spatial distance from the southernmost limit of the range (Spearman rank correlation coefficient rS, all P not significant).
(0.00 MB PDF)

Table S4 Temporal dynamics of occupation of nest sites by ants. Maximum-likelihood estimates [and 95% confidence interval] of annual survival probabilities of host-plant occupancy for Petalomyrmex phylax and Cataulacus mckeyi in six populations ordered from north to south (computed using Mark software). Only the probabilities of continued occupancy of a tree by the same species are presented here. The last row indicates the correlation between population estimates and the spatial distance from the southernmost limit of the range (Spearman rank correlation coefficient rS; ns: not significant, *: P<0.05, **: P<0.01, ***: P<0.001).
(0.00 MB PDF)

Table S5 Geographic variation in P. phylax queen size and potential founding capacity. Within population average (mean±standard deviation) of size and survival of alate female sexuals of Petalomyrmex phylax from 21 populations: head length (HL), head width (HW), partial forewing length (PWL), forewing width (WW), total forewing length (TWL) (mm), dry weight (DW) (in mg), and survival at 60 days under claustral foundation conditions (S60 days). N is the number of individuals, n is the number of colonies. Populations are arranged in descending order according to their map distance from the southernmost known limit of the range of P. phylax. The last row indicates the correlation between population means and the spatial distance from the southernmost limit of the range (Spearman rank correlation coefficient rS; ns: not significant, *: P<0.05, **: P<0.01, ***: P<0.001).
(0.01 MB PDF)

Table S6 Alate female *Petalomyrmex* are larger in the south. Comparison of estimated dry weight (mg) of adult individuals between the two geographically extreme populations (EBO in the south, BOU in the north) according to caste; descriptive statistics and results of ANOVAs. ns: not significant, *: P<0.05, **: P<0.01, ***: P<0.001).
(0.04 MB RTF)
