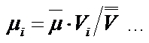# Correction: Range Expansion Drives Dispersal Evolution In An Equatorial Three-Species Symbiosis

**DOI:** 10.1371/annotation/ce7f7827-bc34-49bc-8ad6-e0d87a383a20

**Published:** 2009-07-10

**Authors:** Guillaume Léotard, Gabriel Debout, Ambroise Dalecky, Sylvain Guillot, Laurence Gaume, Doyle McKey, Finn Kjellberg

In the last paragraph of the Materials and Methods subsection "Testing for recent range expansion," the formula for the estimated mutation rate at locus i appears incorrectly. View the correct formula and related text here: 

First, the mutation rate at locus *i* was estimated as