# Supplementary material for: Range Expansion Drives Dispersal Evolution In An Equatorial Three-Species Symbiosis
Source: PLoS One. 2009 Apr 29;4(4):e5377. doi: 10.1371/journal.pone.0005377 (PMC2670579; doi:10.1371/journal.pone.0005377)
Supplement: Table S1 — The study populations: location and measures of genetic diversity. Statistics summarizing within-population genetic variation over 9 microsatellite loci for 19 populations of Leonardoxa a. africana, 12 loci for 21 populations of Petalomyrmex phylax, and 10 loci for 14 populations of Cataulacus mckeyi. Distance is the map distance (in km) from the southern edge of the range of the system. N is the number of diploid individuals genotyped per population; np, number of polymorphic loci; na, mean number of observed alleles; A, mean allelic richness; HE, mean expected heterozygosity; and V, mean variance of allele size. The two last rows indicate the means of values over all populations and the correlation between population means and the map distance from the southernmost limit of the range (Spearman rank correlation coefficient rS, asterisks indicate significant correlation [*: P<0.05, **: P<0.01, ***: P<0.001]). Data for P phylax are from Dalecky et al. [55]. (0.01 MB PDF) [file pone.0005377.s001.pdf]

Table S1. The study populations: location and measures of genetic diversity.

			Leonardoxa a. africana						*Petalomyrmex phylax*						*Cataulacus mckeyi*					
Population	Coordinates	Distance	N	n_p	n_a	A	H_E	V	N	n_p	n_a	A	H_E	V	N	n_p	n_a	A	H_E	V
JFK	03°13'31.80"N // 10°15'04.20"E	85.805							18	12	6.92	6.13	0.658	17.71	8	10	4.8	1.69	0.700	12.11
LA	03°14'27.00"N // 10°13'19.80"E	85.63	22	9	4.33	2.17	0.536	6.85	25	12	6.83	5.71	0.698	12.08						
BOU	03°13'17.32"N // 10°14'56.02"E	85.411	50	9	4.78	2.04	0.486	3.70	66	12	9.5	6.01	0.687	12.12	62	10	8.2	1.58	0.582	7.54
ILO	03°13'22.70"N // 10°14'53.66"E	85.405													5	9	3.7	1.63	0.633	15.25
BM	03°13'15.49"N // 10°14'53.40"E	85.339	80	9	5.22	2.21	0.539	4.05	21	12	7.25	6.02	0.686	10.91	19	10	6.8	1.65	0.646	8.78
DVI	03°12'40.20"N // 10°11'24.00"E	81.037							21	12	7.5	6.3	0.701	12.74						
BP	03°07'21.00"N // 10°00'40.20"E	64.251	23	8	4.56	2.17	0.512	3.06	19	12	8	6.86	0.705	9.01	5	9	3.5	1.63	0.632	9.87
HAN	03°06'58.20"N // 10°01'21.00"E	63.964							16	12	7.42	6.82	0.728	10.66	5	8	3.2	1.54	0.535	9.38
BI	03°01'14.16"N // 10°06'21.37"E	58.081	17	8	3.67	2.00	0.464	3.06												
BSAN	02°59'16.80"N // 10°00'37.80"E	50.185	20	8	3.89	1.92	0.428	2.15	13	12	6.42	6.27	0.71	6.79						
KIEN	02°52'01.20"N // 09°59'07.80"E	36.803	21	7	3.44	1.89	0.415	1.83	27	12	7.17	5.79	0.651	7.43	11	9	4.5	1.51	0.514	11.69
HEVE	02°48'09.60"N // 10°02'10.20"E	33.783	34	9	4.67	1.92	0.416	2.79	19	12	8.42	7.11	0.726	12.71	27	9	6.9	1.61	0.610	10.76
LOBES1	02°51'03.18"N // 09°54'38.34"E	32.329	27	8	3.56	1.89	0.426	2.08												
LOBES2	02°50'49.32"N // 09°54'31.68"E	31.867	52	8	3.89	1.93	0.437	2.16												
NOCA	02°45'51.35"N // 09°53'21.80"E	22.467	19	8	2.89	1.77	0.377	2.07												
VX	02°39'12.00"N // 10°00'49.20"E	21.216							22	12	7.33	6.13	0.708	7.71						
MBO	02°45'13.20"N // 09°53'02.40"E	21.202	32	8	3.89	1.80	0.374	1.47	15	12	6.25	5.85	0.688	6.19						
LB	02°38'39.00"N // 10°00'42.60"E	20.592							18	12	7	6.27	0.674	6.77						
BKA	02°42'05.09"N // 09°51'55.29"E	15.127	13	8	3.11	1.82	0.380	1.74												
KD	02°36'25.20"N // 09°57'48.00"E	14.055							25	12	7.67	6.26	0.665	7.17						
FOR	02°39'28.80"N // 09°51'43.20"E	10.344	19	8	3.44	1.90	0.433	1.90							6	7	3.5	1.48	0.481	8.49
LOL	02°39'24.00"N // 09°51'48.60"E	10.237	39	8	3.67	1.88	0.415	1.88	14	12	6.58	6.22	0.693	6.41						
GRO	02°34'30.00"N // 09°54'06.00"E	6.434							14	12	6.42	6.08	0.712	6.68	6	8	3.4	1.55	0.543	8.21
MAM	02°34'04.20"N // 09°53'48.00"E	5.891	29	8	3.67	1.62	0.297	2.05	22	12	7.5	6.25	0.72	7.4	14	7	5	1.49	0.486	7.50
IPE2	02°36'25.74"N // 09°50'37.00"E	4.508	32	7	3.22	1.70	0.335	1.64												
IPE	02°36'12.60"N // 09°50'31.20"E	4.117							24	12	6.17	5.19	0.577	5.99	7	8	3.1	1.45	0.446	5.50
TM	02°34'02.40"N // 09°50'38.40"E	0.128	18	8	3.44	1.75	0.347	1.88	24	11	6.25	5.03	0.563	5.85	16	8	4.3	1.46	0.460	9.61
TE	02°33'58.80"N // 09°50'40.20"E	0.087							18	10	5.25	4.76	0.533	8.39						
EBO	02°33'58.95"N // 09°50'36.13"E	0	47	9	3.67	1.74	0.348	1.63	34	11	6.42	5.02	0.552	7.18	15	7	3.8	1.44	0.443	8.79
		Mean	31	8.16	3.84	1.90	0.419	2.53	23	11.81	7.06	6	0.668	8.95	15	8.5	4.62	1.55	0.551	9.53
		r_S		0.354	0.636**	0.898***	0.872***	0.829***		0.605**	0.526*	0.392	0.342	0.730***		0.837***	0.337	0.881***	0.881***	0.433

Statistics summarizing within-population genetic variation over 9 microsatellite loci for 19 populations of *Leonardoxa a. africana*, 12 loci for 21 populations of *Petalomyrmex phylax*, and 10 loci for 14 populations of *Cataulacus mckeyi*. Distance is the map distance (in km) from the southern edge of the range of the system. N is the number of diploid individuals genotyped per population; n_p, number of polymorphic loci; n_a, mean number of observed alleles; A, mean allelic richness; H_E, mean expected heterozygosity; and V, mean variance of allele size. The two last rows indicate the means of values over all populations and the correlation between population means and the map distance from the southernmost limit of the range (Spearman rank correlation coefficient r_S, asterisks indicate significant correlation [*: $P < 0.05$, **: $P < 0.01$, ***: $P < 0.001$]). Data for *P phylax* are from Dalecky et al. [55].
