# Supplementary material for: Range Expansion Drives Dispersal Evolution In An Equatorial Three-Species Symbiosis
Source: PLoS One. 2009 Apr 29;4(4):e5377. doi: 10.1371/journal.pone.0005377 (PMC2670579; doi:10.1371/journal.pone.0005377)
Supplement: Table S2 — Size at which half the trees had reached sexual maturity. Estimated values [and 95% confidence interval] across trees per population and over all individuals of basal trunk diameter and tree height at which the probability of flowering is 0.50 based on logistic regressions. Populations are arranged in descending order according to their geographical distance from the southernmost known limit of the range of the system (these distances are given in Table S1). N is the total number of trees sampled, m is the number of sexually mature trees. The last row indicates the correlation between population estimates and the spatial distance from the southernmost limit of the range (Spearman rank correlation coefficient rS, all P not significant). (0.00 MB PDF) [file pone.0005377.s002.pdf]

Table S2. Size at which half the trees had reached sexual maturity.

Population	*N* / *m*	Basal trunk diameter (mm)	Tree height (m)
BM	71 / 27	23 [19–29]	1.7 [1.4–2.2]
BP	54 / 22	17 [15–19]	1.7 [1.4–2.2]
LOBES2	60 / 20	26 [20–36]	2.5 [1.8–4.1]
MBO	62 / 24	19 [16–23]	2.0 [1.6–2.7]
LOL	41 / 20	25 [21–29]	2.4 [1.8–3.1]
EBO	124 / 89	21 [18–24]	1.5 [1.2–1.7]
**Overall**	**402 / 202**	**22 [20–24]**	**1.7 [1.5–1.9]**
	r_S	-0.086 ns	0.058 ns

Estimated values [and 95% confidence interval] across trees per population and over all individuals of basal trunk diameter and tree height at which the probability of flowering is 0.50 based on logistic regressions. Populations are arranged in descending order according to their geographical distance from the southernmost known limit of the range of the system (these distances are given in Table S1). *N* is the total number of trees sampled, *m* is the number of sexually mature trees. The last row indicates the correlation between population estimates and the spatial distance from the southernmost limit of the range (Spearman rank correlation coefficient r_S, all *P* not significant).
