# Supplementary material for: Range Expansion Drives Dispersal Evolution In An Equatorial Three-Species Symbiosis
Source: PLoS One. 2009 Apr 29;4(4):e5377. doi: 10.1371/journal.pone.0005377 (PMC2670579; doi:10.1371/journal.pone.0005377)
Supplement: Table S3 — Tree investment in the feeding of ants. Mean (±standard deviation) values across trees per population of number of (1) observed nectaries per leaf and of (2) produced nectaries per basal leaflet. Populations are arranged in descending order according to their geographical distance from the southernmost known limit of the range of the system (these distances are given in Table S1). N is the number of trees sampled. The last row indicates the correlation between population means and the spatial distance from the southernmost limit of the range (Spearman rank correlation coefficient rS, all P not significant). (0.00 MB PDF) [file pone.0005377.s003.pdf]

**Table S3.** Tree investment in the feeding of ants.

| Population | *N* | (1) Number of observed nectaries per leaf | (2) Number of produced nectaries per basal leaflet |
|---|---|---|---|
| JFK | 10 | 13.94 (± 1.57) | 3.11 (± 0.30) |
| LA | 10 | 12.84 (± 1.27) | 3.00 (± 0.31) |
| BOU | 30 | 11.01 (± 2.05) | 2.92 (± 0.35) |
| BM | 10 | 13.08 (± 1.63) | 3.09 (± 0.31) |
| BP | 10 | 12.95 (± 1.71) | 3.32 (± 0.24) |
| HEVE | 10 | 11.57 (± 1.87) | 3.10 (± 0.22) |
| MBO | 10 | 12.52 (± 2.00) | 3.08 (± 0.27) |
| LOL | 10 | 13.61 (±2.59) | 3.21 (± 0.64) |
| IPE | 10 | 13.66 (± 0.96) | 3.12 (± 0.24) |
| TM | 12 | 12.67 (± 1.77) | 3.02 (± 0.30) |
| TE | 18 | 12.41 (± 1.63) | 3.18 (± 0.38) |
| EBO | 21 | 13.21 (± 2.13) | 3.35 (± 0.45) |
| | $r_S$ | 0.021 ns | -0.497 ns |

Mean (± standard deviation) values across trees per population of number of (1) observed nectaries per leaf and of (2) produced nectaries per basal leaflet. Populations are arranged in descending order according to their geographical distance from the southernmost known limit of the range of the system (these distances are given in Table S1). *N* is the number of trees sampled. The last row indicates the correlation between population means and the spatial distance from the southernmost limit of the range (Spearman rank correlation coefficient $r_S$, all *P* not significant).
