# Supplementary material for: Range Expansion Drives Dispersal Evolution In An Equatorial Three-Species Symbiosis
Source: PLoS One. 2009 Apr 29;4(4):e5377. doi: 10.1371/journal.pone.0005377 (PMC2670579; doi:10.1371/journal.pone.0005377)
Supplement: Table S4 — Temporal dynamics of occupation of nest sites by ants. Maximum-likelihood estimates [and 95% confidence interval] of annual survival probabilities of host-plant occupancy for Petalomyrmex phylax and Cataulacus mckeyi in six populations ordered from north to south (computed using Mark software). Only the probabilities of continued occupancy of a tree by the same species are presented here. The last row indicates the correlation between population estimates and the spatial distance from the southernmost limit of the range (Spearman rank correlation coefficient rS; ns: not significant, *: P<0.05, **: P<0.01, ***: P<0.001). (0.00 MB PDF) [file pone.0005377.s004.pdf]

Table S4. Temporal dynamics of occupation of nest sites by ants.

Population	*C. mckeyi*	*P. phylax*
BOU	0.76 [0.28-0.96]	0.93 [0.73-0.97]
BM	0.69 [0.36-0.90]	0.90 [0.65-0.95]
HEVE	0.71 [0.42-0.89]	0.96 [0.75-0.98]
TM	0.60 [0.45-0.73]	0.87 [0.84-0.88]
TE	0.33 [0.04-0.85]	0.92 [0.85-0.94]
EBO	0.56 [0.42-0.68]	0.91 [0.79-0.94]
r_S	0.886*	0.257 ns

Maximum-likelihood estimates [and 95% confidence interval] of annual survival probabilities of host-plant occupancy for *Petalomyrmex phylax* and *Cataulacus mckeyi* in six populations ordered from north to south (computed using Mark software). Only the probabilities of continued occupancy of a tree by the same species are presented here. The last row indicates the correlation between population estimates and the spatial distance from the southernmost limit of the range (Spearman rank correlation coefficient r_S; ns: not significant, *: $P < 0.05$, **: $P < 0.01$, ***: $P < 0.001$).
