# Supplementary material for: Range Expansion Drives Dispersal Evolution In An Equatorial Three-Species Symbiosis
Source: PLoS One. 2009 Apr 29;4(4):e5377. doi: 10.1371/journal.pone.0005377 (PMC2670579; doi:10.1371/journal.pone.0005377)
Supplement: Table S5 — Geographic variation in P. phylax queen size and potential founding capacity. Within population average (mean±standard deviation) of size and survival of alate female sexuals of Petalomyrmex phylax from 21 populations: head length (HL), head width (HW), partial forewing length (PWL), forewing width (WW), total forewing length (TWL) (mm), dry weight (DW) (in mg), and survival at 60 days under claustral foundation conditions (S60 days). N is the number of individuals, n is the number of colonies. Populations are arranged in descending order according to their map distance from the southernmost known limit of the range of P. phylax. The last row indicates the correlation between population means and the spatial distance from the southernmost limit of the range (Spearman rank correlation coefficient rS; ns: not significant, *: P<0.05, **: P<0.01, ***: P<0.001). (0.01 MB PDF) [file pone.0005377.s005.pdf]

Table S5. Geographic variation in *P. phylax* queen size and potential founding capacity.

Population	HL (*N/n*)	HW (*N/n*)	PWL (*N/n*)	WW (*N/n*)	TWL (*N/n*)	DW (*N/n*)	$S_{60\ days}$ (*N/n*)
JFK	0.927 ± 0.033 (17/7)	1.023 ± 0.032 (17/7)	3.697 ± 0.148 (7/2)	1.833 ± 0.067 (7/2)	5.494 ± 0.206 (7/2)	1.675 ± 0.282 (20/17)	0.083 (12/7)
LA	0.949 ± 0.021 (22/5)	1.025 ± 0.027 (22/5)	3.729 ± 0.089 (5/2)	1.845 ± 0.049 (5/2)	5.605 ± 0.135 (5/2)	1.657 ± 0.307 (19/19)	0.083 (12/8)
BOU	0.929 ± 0.027 (376/53)	1.017 ± 0.030 (221/46)	3.681 ± 0.113 (59/9)	1.861 ± 0.050 (59/9)	5.477 ± 0.147 (50/8)	1.824 ± 0.241 (21/20)	0.200 (15/10)
BM	0.924 ± 0.021 (31/26)	1.014 ± 0.025 (31/26)	3.581 ± 0.140 (4/4)	1.838 ± 0.067 (3/3)	5.346 ± 0.235 (4/4)	1.739 ± 0.308 (14/9)	–
DVI	0.922 ± 0.023 (49/10)	1.031 ± 0.029 (49/10)	3.500 ± 0.087 (19/6)	1.816 ± 0.032 (19/6)	5.349 ± 0.100 (19/6)	–	–
BP	0.949 ± 0.022 (30/2)	1.029 ± 0.025 (30/2)	3.537 ± 0.083 (11/1)	1.820 ± 0.044 (10/1)	5.344 ± 0.115 (10/1)	1.531 ± 0.260 (9/6)	0.250 (12/8)
HAN	0.945 ± 0.019 (14/3)	1.030 ± 0.024 (14/3)	3.800 (1/1)	1.892 (1/1)	5.637 (1/1)	1.733 ± 0.309 (4/4)	–
BSAN	0.939 ± 0.021 (13/3)	1.042 ± 0.031 (13/3)	3.567 ± 0.082 (9/2)	1.812 ± 0.029 (9/2)	5.429 ± 0.115 (9/2)	–	–
KIE	0.961 ± 0.032 (29/4)	1.055 ± 0.038 (29/4)	3.821 ± 0.032 (2/1)	1.856 ± 0.001 (2/1)	5.754 ± 0.065 (2/1)	2.185 ± 0.407 (3/1)	–
HEVE	0.974 ± 0.027 (57/14)	1.060 ± 0.026 (57/14)	3.789 ± 0.081 (30/3)	1.873 ± 0.030 (30/3)	5.659 ± 0.096 (29/3)	2.269 ± 0.347 (10/9)	0.167 (12/7)
VX	0.980 ± 0.037 (10/4)	1.079 ± 0.029 (10/4)	3.620 (1/1)	1.793 (1/1)	5.448 (1/1)	–	–
MBO	1.016 ± 0.018 (34/8)	1.088 ± 0.029 (34/8)	3.861 ± 0.098 (29/5)	1.931 ± 0.037 (29/5)	5.828 ± 0.129 (28/5)	2.430 ± 0.231 (20/12)	0.267 (15/9)
LB	1.024 ± 0.029 (13/4)	1.116 ± 0.030 (13/4)	–	–	–	2.610 ± 0.305 (9/6)	–
KD	1.054 ± 0.025 (36/17)	1.135 ± 0.029 (36/17)	–	–	–	–	–
LOL	1.056 ± 0.021 (20/5)	1.146 ± 0.024 (20/5)	–	–	–	2.461 ± 0.389 (20/15)	0.294 (17/8)
GRO	1.065 ± 0.028 (21/12)	1.152 ± 0.020 (21/12)	4.144 ± 0.055 (2/2)	2.039 ± 0.084 (2/2)	6.075 ± 0.058 (2/2)	2.561 ± 0.307 (6 2)	–
MAM	1.061 ± 0.028 (91/15)	1.146 ± 0.021 (91/15)	4.029 ± 0.086 (61/6)	2.023 ± 0.071 (61/6)	5.979 ± 0.117 (42/6)	–	–
IPE	1.065 ± 0.021 (23/12)	1.162 ± 0.022 (23/12)	4.115 ± 0.069 (4/4)	2.036 ± 0.013 (4/4)	6.137 ± 0.135 (4/4)	2.799 ± 0.387 (24/18)	0.333 (18/9)
TM	1.049 ± 0.030 (23/5)	1.152 ± 0.027 (23/5)	4.046 ± 0.097 (11/2)	1.996 ± 0.069 (11/2)	6.048 ± 0.117 (11/2)	2.538 ± 0.413 (20/19)	0.231 (13/10)
TE	1.050 ± 0.028 (20/11)	1.152 ± 0.023 (20/11)	4.039 ± 0.104 (11/5)	2.058 ± 0.051 (11/5)	6.047 ± 0.129 (11/5)	–	–
EBO	1.053 ± 0.023 (107/36)	1.154 ± 0.028 (107/36)	3.995 ± 0.078 (49/11)	1.998 ± 0.045 (49/11)	5.959 ± 0.120 (48/11)	2.586 ± 0.344 (20/19)	0.400 (20/9)
r_S	-0.877***	-0.964***	-0.746***	-0.705***	-0.765***	-0.879***	-0.827**

Within population average (mean ± standard deviation) of size and survival of alate female sexuals of *Petalomyrmex phylax* from 21 populations: head length (HL), head width (HW), partial forewing length (PWL), forewing width (WW), total forewing length (TWL) (mm), dry weight (DW) (in mg), and survival at 60 days under claustral foundation conditions ($S_{60\ days}$). *N* is the number of individuals, *n* is the number of colonies. Populations are arranged in descending order according to their map distance from the southernmost known limit of the range of *P. phylax*. The last row indicates the correlation between population means and the spatial distance from the southernmost limit of the range (Spearman rank correlation coefficient r_S; ns: not significant, *: $P < 0.05$, **: $P < 0.01$, ***: $P < 0.001$).
