# Supplementary material for: Range Expansion Drives Dispersal Evolution In An Equatorial Three-Species Symbiosis
Source: PLoS One. 2009 Apr 29;4(4):e5377. doi: 10.1371/journal.pone.0005377 (PMC2670579; doi:10.1371/journal.pone.0005377)
Supplement: Table S6 — Alate female Petalomyrmex are larger in the south. Comparison of estimated dry weight (mg) of adult individuals between the two geographically extreme populations (EBO in the south, BOU in the north) according to caste; descriptive statistics and results of ANOVAs. ns: not significant, *: P<0.05, **: P<0.01, ***: P<0.001). (0.04 MB RTF) [file pone.0005377.s006.doc]

**Table S6.** Alate female *Petalomyrmex* are larger in the south

Comparison of estimated dry weight (mg) of adult individuals between the two geographically extreme populations (EBO in the south, BOU in the north) according to caste; descriptive statistics and results of ANOVAs. ns: not significant, *: *P* < 0.05, **: *P* < 0.01, ***: *P* < 0.001).

| Caste |  | Adult alate females | | |  |  | Adult males | | |  |  | Adult workers | | |  |
| --- | --- | --- | --- | --- | --- | --- | --- | --- | --- | --- | --- | --- | --- | --- | --- |
| Population |  | *n* | Mean ± sd | |  |  | *N* | Mean ± sd | |  |  | *n* | Mean ± sd | |  |
| EBO (south) |  | 30 | 1.926 ± 0.585 | |  |  | 14 | 0.279 ± 0.048 | |  |  | 101 | 0.065 ± 0.017 | |  |
| BOU (north) |  | 20 | 0.834 ± 0.202 | |  |  | 16 | 0.259 ± 0.033 | |  |  | 89 | 0.058 ± 0.016 | |  |
| Overall |  | 50 | 1.489 ± 0.715 | |  |  | 30 | 0.268 ± 0.041 | |  |  | 190 | 0.062 ± 0.017 | |  |
| ANOVA results |  | Adult alate females | | |  |  | Adult males | | |  |  | Adult workers | | |  |
| Explanatory variable |  | df | MS | *F* |  |  | df | MS | *F* |  |  | df | MS | *F* |  |
| Population |  | 1 | 11.3611 | **29.59***** |  |  | 1 | 0.0004 | **0.28ns** |  |  | 1 | 0.0020 | **0.98ns** |  |
| Colony (population) |  | 14 | 0.3839 | 2.44* |  |  | 7 | 0.0015 | 0.93ns |  |  | 17 | 0.0020 | 17.29*** |  |
| Error |  | 34 | 0.1572 |  |  |  | 21 | 0.0017 |  |  |  | 171 | 0.0001 |  |  |
